# Supplementary material for: Extracellular Matrix Biomimetic Hydrogels, Encapsulated with Stromal Cell-Derived Factor 1, Improve the Composition of Foetal Tissue Grafts in a Rodent Model of Parkinson’s Disease
Source: Int J Mol Sci. 2022 Apr 22;23(9):4646. doi: 10.3390/ijms23094646 (PMC9101815; doi:10.3390/ijms23094646)
Supplement: Supplementary file 1 [file ijms-23-04646-s001.zip › ijms-1666325-supplementary.pdf]

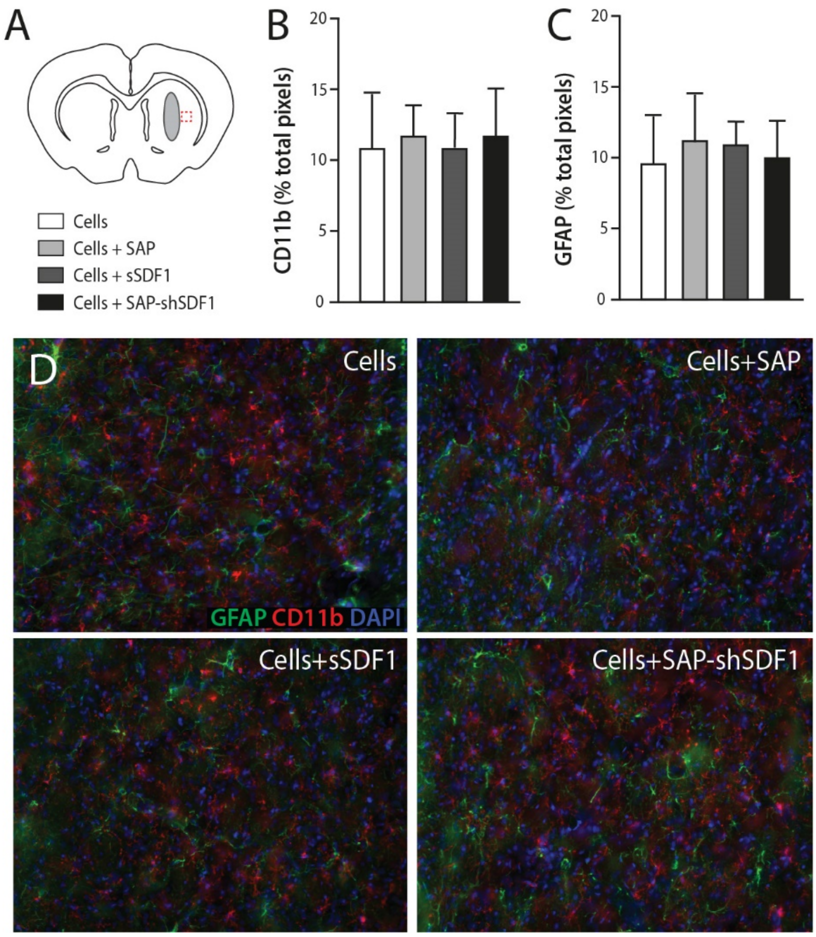

Supplementary Figure S1 – *SDF1 functionalised hydrogels do not evoke a host inflammatory response.* (A) Schematic illustration showing a coronal section of the mouse brain, depicting the graft (grey) and the sampling site (red box) lateral to the graft-host border for assessment of the host inflammatory response to. (B) Density of CD11b microglia and (C) GFAP+ reactive astrocytes (expressed as the percentage of immunoreactive pixels) was unchanged by the presence of the SAP hydrogel and/or SDF1 protein. (D) Representative photomicrographs of GFAP+ astrocytes and CD11b+ microglia taken at the graft-host border from the 4 grafting groups. Data represents Mean $\pm$ SEM, n=4/group.
